# Generation of Skin Organoids: Potential Opportunities and Challenges

**DOI:** 10.3389/fcell.2021.709824

**Published:** 2021-11-04

**Authors:** Hui Sun, Yi-Xuan Zhang, Yu-Mei Li

**Affiliations:** ^1^ Institute of Regenerative Medicine, Affiliated Hospital of Jiangsu University, Jiangsu University, Zhenjiang, China; ^2^ Department of Dermatology, Affiliated Hospital of Jiangsu University, Jiangsu University, Zhenjiang, China; ^3^ School of Medicine, Jiangsu University, Zhenjiang, China

**Keywords:** skin organoid, skin stem cell niches, 3D culture, single-cell RNA sequencing, skin generation

## Abstract

Although several types of human skin substitutes are currently available, they usually do not include important skin appendages such as hair follicles and sweat glands, or various skin-related cells, such as dermal adipocytes and sensory neurons. This highlights the need to improve the *in vitro* human skin generation model for use as a tool for investigating skin diseases and as a source of cells or tissues for skin regeneration. Skin organoids are generated from stem cells and are expected to possess the complexity and function of natural skin. Here, we summarize the current literatures relating to the “niches” of the local skin stem cell microenvironment and the formation of skin organoids, and then discuss the opportunities and challenges associated with multifunctional skin organoids.

## Introduction

The skin is a very complex organ that comprises various stem cell populations as well as numerous other cell types (Clevers et al., 2014; Chen et al., 2018). The skin plays an essential role in maintaining the stability of the body’s internal environment, protecting the body from daily attrition, and regulating the body’s temperature and perception. The outer boundary of the skin, known as the epidermis, is maintained by epidermal stem cells that reside in the basal layer, while the dermis, the layer under the epidermis, is rich in dermal fibroblasts that produce extracellular matrix components, such as collagen and elastic fibers, that give the skin its elasticity. Under the dermis lies subcutaneous fat, which functions as padding, insulation, and energy storage (Takeo et al., 2015; Gravitz, 2018). However, the current *in vitro*-generated skin models lack many of the normal and necessary skin structures, so we should find new alternative cells and functional models for skin diseases and regeneration.

The continuous and rapid development of stem cell biology can provide new insights into the basic biology of human diseases, thereby driving innovation in the diagnosis and treatment of a variety of conditions. Human pluripotent stem cells (hPSCs), including human embryonic stem cells (hESCs) and human induced pluripotent stem cells (hiPSCs), have become a valuable tool for modeling human diseases, complementing traditional animal research models (Takahashi and Yamanaka, 2006; Takahashi et al., 2007; Shi et al., 2017). Under specific induction conditions, hPSCs can differentiate into any cell or tissue type of the human body. They can also be used to generate three-dimensional (3D) *in vitro* models, i.e., organoids, containing various cell types that can be used for modeling organogenesis and developmental disorders (Dutta et al., 2017; Rossi et al., 2018). Skin organoids are derived from hPSCs and can self-assemble to form an organized, skin-like structure composed of skin progenitor cells and hair follicles resembling fetal skin (Lee et al., 2018; Kim and Ju, 2019; Lee et al., 2020).

In this review, we first evaluate recent advances in skin formation in the field of stem cells and regenerative medicine. Then, we discuss the origins of skin cells and skin organoid culture systems and offer feasible suggestions for future directions and methods to generate ideal skin organoids using the latest technologies, such as 3D culture. Despite their tremendous promise, current skin organoid models still have limitations. Therefore, in this review, we also aim to provide an impartial view of the opportunities and challenges relating to skin organoids. Only when current models are fully understood can skin organoids help shed light on our understanding of human skin biology and skin diseases.

## Skin Development and Microenvironment

**FIGURE 1:**
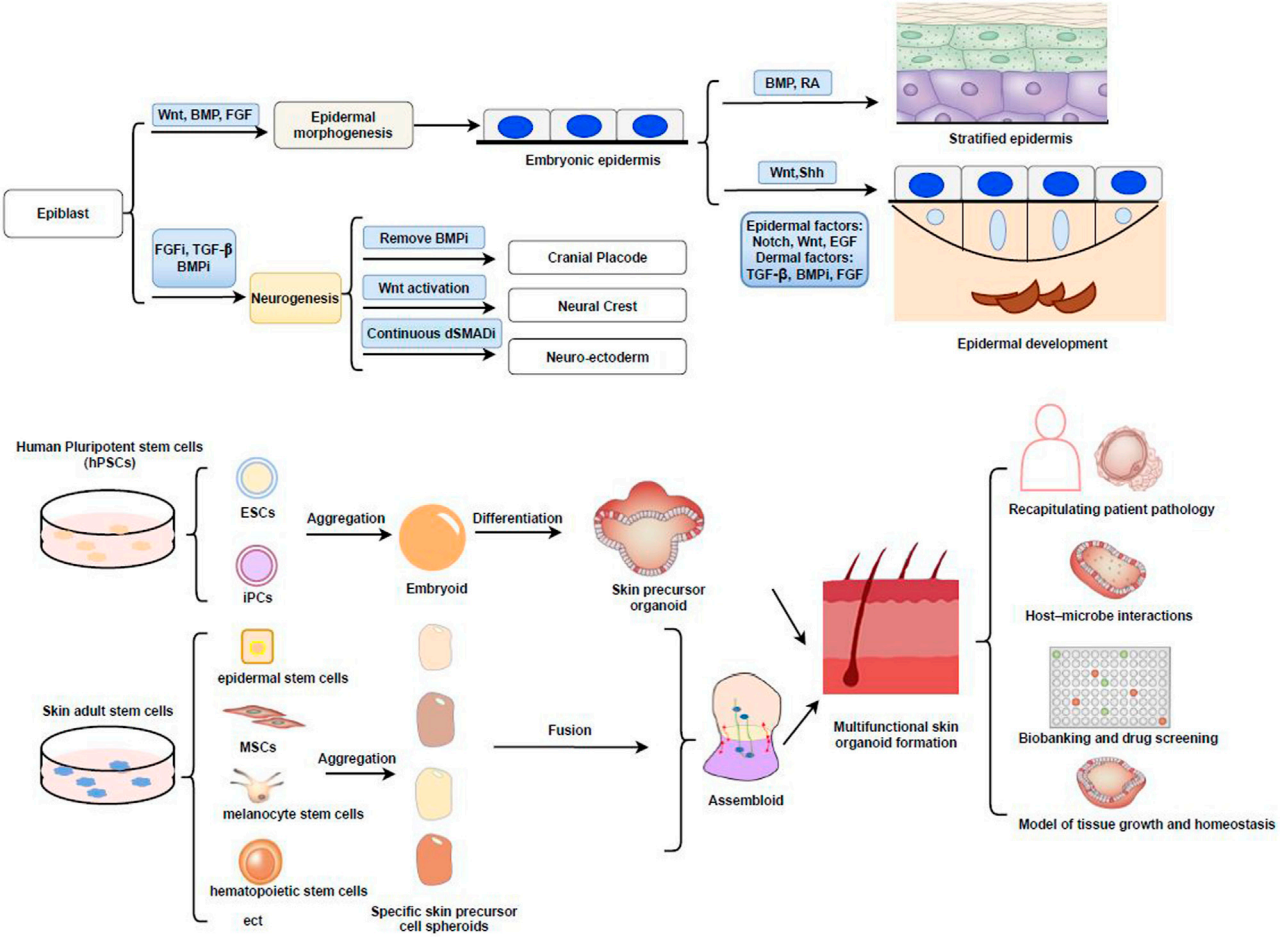
**(A)** Early signaling of skin development. Wnt signaling blocks the ability of early ectodermal progenitor cells, allowing them to respond to BMP signaling and adopt an epidermal fate. Representation of the border model and important signaling pathways such as mall molecules termed as dual SMAD inhibition (dSMADi) that inhibit the BMP and TGFβ signaling pathways, respectively and influence particular cell fates in neurogenesis as well as CP, NC, NE. While Wnt/Shh activating signals produce the hair placode and additional dermal messages further instruct the placodes to make the hair follicle. **(B)** Schematic illustration of skin organoid formation and its application. This figure summarizes how integration of skin organoid technology with PSC/ASC approaches (including EB formation and fusion assembloids with different skin precursor cells) allowed the understanding of various applications in skin diseases. Cranial placode, CP; dual SMAD inhibition, dSMADi; Embryoid-body, EB; Neural crest, NC; Neuroectoderm, NE; Retinoic acid, RA.

Different types of skin-resident cells perform different functions; thus, we must first fully understand the mechanisms involved in skin development as well as the microenvironment. Importantly, multiple types of skin stem cells, such as epidermal stem cells, as well as other types of progenitor cells that reside in the skin and its appendages (e.g., hair bulbs and sweat glands), are required for the development, continuous renewal, and regeneration of the body’s skin, activities that are controlled by the “niches” within the local stem cell microenvironment (Fuchs, 2007; Yin et al., 20132011; Mahmoudi and Brunet, 2012). With the development of genetic and imaging tools, our understanding of the relationship between skin stem cells and their progeny, as well as the interaction between skin stem cells and their niches, has increased over recent years (Gravitz, 2018). Wnt signaling emanating from the niches has been proposed to act as a cue for stem cell self-renewal in a variety of mammalian tissues (Clevers et al., 2014) *hai*(Figure 1A). At the same time, lineage analysis demonstrated heterogeneity among the cells in stem or progenitor cells are stochastic. In addition, stem cells and their downstream progenitors exploit the full advantages of their own environment according to detailed areas as niches (Ito et al., 2007; Yang et al., 2017). Therefore, skin stem cells, their niches, and associated signals are essential for skin development and differentiation. In addition, the maintenance and function of adult skin stem cells require different microenvironments within the niches, including the presence of a variety of allogeneic cells and associated stem cell lineages (Lane et al., 2014; Gonzales and Fuchs, 2017).

The skin originates from two major tissue types, namely, the ectoderm (epidermis) and mesenchyme (dermis, hypodermis, and mesodermal connective tissue). Sensory nerve endings and melanocytes (neural crest) are also present in the skin (Quarto et al., 2010). In the early embryo, Wnt/β-catenin signaling plays a key role in skin development (Siebel and Lendahl, 2017; Lim and Nusse, 2013). During gastrulation, the ectoderm invaginates along the midline for development, and then the ectodermal cells proliferate and migrate downwards from the center (Tchieu et al., 2017). Three primary cell layers (germ layers) are formed in the gastrula: ectoderm, mesoderm, and endoderm. The ectodermal layer lies on the surface of the embryo, and whether or not the ectoderm develops into skin is dependent on Wnt signaling (Fuchs, 2007). In the absence of FGF signaling, ectodermal cells express bone morphogenetic protein (BMP) and develop into epidermis (Tchieu et al., 2017). Additionally, in the absence of Wnt signaling, FGF signaling is activated, BMP expression is weakened, and ectodermal cells can also adopt a neural crest fate (Figure 1A) (Ji et al., 2019; Girgin et al., 2021). By precisely modulating the activities of the FGF, BMP, Wnt, and transforming growth factor β (TGF-β) pathways using different substrates and a chemically defined medium, reproducible derivations of neuroectoderm, neural crest, cranial placode, and non-neural ectoderm can be achieved (Tchieu et al., 2017) (Figure 1A).

During early skin development, within several days, cell divisions become first oblique, and then more perpendicular, leading to asymmetric fates, as well as the differentiation and stratification of the epidermis (Matsumura et al., 2021). When morphogenesis is complete, progenitor cells of the epidermis and appendages maintain their contact with the basement membrane and express markers that identify them as keratinocytes (Green et al., 2003).

To conclude, skin formation is a multistep process, involving the growth of the dermis and epidermis, as well as skin appendages. Skin appendages such as hair placodes form from the congregation of Wnt^high^ cells in the basal layer (Gonzales and Fuchs, 2017). These cells begin to divide perpendicular to the basement membrane, leading to asymmetrically fated daughters. Wnt^high^ cells produce sonic hedgehog (Shh), but only neighboring cells can respond to this signal. Shh signaling induces the underlying mesenchyme to organize into a dermal condensate and simultaneously produce BMP inhibitors. Shh signaling also prompts the covering daughter cells that lose contact with the basement membrane to dampen Wnt signaling (Wnt^low^) and divide symmetrically. These Wnt^low^ daughters will generate the outer root sheath, which can develop a niche (bulges) of stem cells, while the Wnt^high^ daughters may generate the inner root sheath or hair shaft (Hsu and Fuchs, 2012; Gonzales and Fuchs, 2017).

Knowledge about the early stages of human melanocyte and skin appendage fate specification is limited to basic histological studies (Xie et al., 2016; Wang et al., 2020). Lineage tracing using single-cell sequencing has identified important differences in regenerative and developmental strategies among skin stem cells (e.g., bulge stem cells) or normal cells (e.g., adipocytes, melanocytes) (Guerrero-Juarez et al., 2019; Kim et al., 2020; Solé-Boldo et al., 2020). Epithelial and mesenchymal structures form from several types of fate-restricted progenitors. The skin appendage, a functional mini organ, develops in a dynamic environment influenced by a variety of molecular signals (Lim and Nusse, 2013).

Different signaling patterns at different stages of development ensure the growth and progression of the corium and its derivatives (Fuchs, 2007; Hsu and Fuchs, 2012). Every step of epidermal development is closely related to the development of the dermis and the substratum, while differences in the dermis can lead to regional heterogeneity of the overlying epidermis (Zhu et al., 20141004). A variety of cells interact during skin formation (Figure 1A).

## Sources of Skin Cells and the Generation of Skin Organoids

The management of skin defects, both local and full thickness, remains a considerable clinical challenge. The current treatment option consists mainly of medium-thickness skin grafts, but this is constrained by donor site limitations (Meuli et al., 2019). Cell transplantation is a novel treatment that involves isolating cells from small skin biopsies and seeding them in collagen hydrogels (Meuli et al., 2019; Shpichka et al., 2019). While new skin substitutes provide safe coverage of skin defects, traditional skin tissue engineering methods reduce the complexity of skin tissue to two main parts (epidermis and dermis), which does not allow the function of the patient’s skin to be reproduced (Weng et al., 2020). Meanwhile, keratinocyte culture *in vitro* is time-consuming and labor-intensive, and the generated skin substitutes lack appendages (Tjin et al., 2020; Weng et al., 2020).

Stem cells are the foundation of all mammalian life. The establishment of 3D culture systems (or organoids, where an organ is in a dish) is revolutionizing the way human biology is studied (Kaluthantrige Don and Huch, 2021). Organoids are generated *via* the 3D culture of isolated tissue progenitor cells or PSCs and need an appropriate environmental background for development (Lei et al., 2017).

Stem cells are the main source of cells for the construction of organoids (Yin et al., 2016). There are two main types of stem cells. One is adult stem cells (ASCs), with each organ having its own specialized ASCs, which usually reside in “niches” that provide distinct microenvironments for stem cell maintenance and function (Mahmoudi and Brunet, 2012). The skin contains a variety of ASCs, including epidermal, hair follicle, dermal mesenchymal (MSCs), melanocyte, endothelial, and hematopoietic stem cells, among others (Figure 1B) (Hsu et al., 2014). These ASCs can replace tissue lost through daily attrition, trauma, or disease. Numerous studies have reported on ASC-derived skin organoids, such as those related to the epidermis, sweat glands, and hair follicles (Higgins et al., 2013; Boonekamp et al., 2019; Diao et al., 2019). In addition, the other type is PSCs, including ESCs and iPSCs (Takahashi and Yamanaka, 2006; Mora et al., 2017). Recent studies have shown that iPSCs can be well controlled and optimized to generate embryoid bodies and then differentiate into fibroblasts and keratinocytes under specific condition in the early stage (Kim et al., 2018). However, the derivation of iPSCs by ectopic expression of core pluripotency factors adds the concerns for PSC tumorigenesis (Lee et al., 2013). Cord blood mononuclear cells (CBMCs) have also become a source of replacement cells, and CBMC-derived iPSCs have been differentiated into keratinocytes and fibroblasts, as well as into 3D skin organoids. The derived keratinocytes and fibroblasts have characteristics similar to those of the original cell lines (Kim and Ju, 2019).

Recently, Lei et al. demonstrated *in vitro* skin organoids to form skin with hairs from dissociated cells (Lei et al., 2017), while Lee et al. reported a skin organoid culture system that produces complex skin from human PSCs (iPS/ES) (Table 1) (Lee et al., 2020). Within spherical cell aggregates, non-neuroectodermal and neural crest cells were induced by the stepwise regulation of the TGF-β and FGF signaling pathways. During the 45-months incubation period, the existing skin organoid had developed stratified epidermis, fat-rich dermis, and pigmented hair follicles with sebaceous glands. A network of sensory neurons and Schwann cells that formed neuro-like bundles could also be seen. Additionally, when transplanted into nude mice, these organoids formed flat, hairy, and almost completely natural skin.

**TABLE 1:** Overview of current skin organoid methods. A summary of important parameters in skin organoids, including types of stem cells, species, skin organoid identity, source of starting materials, intrinsic patterning or extrinsic signaling molecules, extracellular scaffold and/or bioreactor, whether to use high-throughput sequencing and special considerations for skin modeling. ASCs, Adult stem cells; BMP4, Bone Morphogenetic Protein 4; CDB, Clustering-dependent embryoid body; DP, Dermal papilla; EBs, Embryonic bodies; EEM, Epidermal expansion medium; PSCs, Pluripotent stem cells; RA, Retinoic Acid; TGF-βi, TGF-β signaling inhibitor; 3D, three-dimensional;

Types of stem cells	Species	Organoid identity	Starting cells	Intrinsic patterning or extrinsic signaling molecules	Extracellular scaffold and/or bioreactor	High-throughput analysis	Special considerations for skin modeling	References
ASCs	Mouse	Murine epidermal organoid	Murine epidermal stem cells	EEM, Noggin, R-spondin 1, Forskolin, and FGF1	Basement membrane extract (Cultrex)	Whole mRNA transcriptome analysis	Serving as a model to study adult epidermal homeostasis and disease *in vitro*	Boonekamp et al. (2019)
	Human	Organoid model of dermal papilla of human hair follicle	DP fibroblasts	Contextual human hair induction assay using dermal papillae implanted into human skin	Cultured as 3D dermal papilla fibroblast spheroids	Affymetrix microarray analysis	When grown as spheroids, human dermal papilla cells induce *de novo* hair follicles in skin	Higgins et al. (2013)
	Mouse	Sweat gland organoid	Sweat gland cells	EGF, bFGF, EDA, TGF-βi, FSK, BMP4	Matrigel tailored for sweat glands formed epithelial organoids	NA	A new strategy for regenerating functional sweat gland-like structures	Diao et al. (2019)
	Mouse	3D skin organoid	Newborn mouse skin cells	PKC inhibition	Epidermal cells and dermal cells in one droplet	Whole transcriptome RNA-seq	Functional perturbation with inhibitors of the key molecules at each phase-transition stage can suppress or accelerate the phase-transition process to form skin	Lei et al. (2017)
				IGF2, VEGF, Wnt3a, Wnt10b, and MMP14				
PSCs	Human	Induced pluripotent stem cell-derived skin organoid	iPSC-derived fibroblasts	RA, BMP4, and EGF.	Embryonic bodies	NA	Using CBMC-iPSCs as a novel tool for *in vitro* and *in vivo* dermatological research	Kim et al. (2018), Kim and Ju (2019)
			iPSC-derived keratinocytes		Coating with type I collagen for fibroblasts			
					Coating with type IV collagen			
					Transwell plate			
	Mouse	Integumentary organ system (IOS)	iPSCs	A mitomycin C–treated SNLP feeder layer	CDB	NA	Establishing a novel non-animal assay system, including appendages such as hair follicles and sebaceous glands, for cosmetics and quasi-drug testing	Takagi et al. (2016), Toyoshima et al. (2019)
				Wnt10b a novel *in vivo* transplantation model				
	Mouse	PSC-derived skin organoid	ESCs iPSCs	TGF-β inhibitor	Matrigel	NA	Studying minimal cellular and microenvironmental requirements for hair follicle induction, evaluating hair follicle growth-promoting/inhibitory drugs, or modeling skin diseases	Lee et al. (2018)
				BMP4				
				FGF-2				
				BMP inhibitor				
	Human	Hair-bearing human skin organoid	ESCs iPSCs	BMP4 bFGF	Matrigel	Single-cell RNA-seq to gain insight into the cell lineages arising in skin organoids	Establishing a model for investigating the cellular dynamics of developing human skin and its appendages	Lee et al. (2020), Lee and Koehler (2021)
				TGFβ inhibitor	Orbital shaker			
				BMP inhibitor				

Skin organoids can produce skin appendages, especially hair follicles, which grow radially and receive innervation from sensory neurons (Lee et al., 2020). Additionally, the hair follicles of skin organoids can reach a level of maturity that is roughly equivalent to second-trimester fetal hair and exhibit the cellular components required for further maturation. However, long-term culture is needed to determine whether xenografted skin organoid-derived follicles can convert to a terminal hair fate or maintain a growth cycle *in vivo* (Lee et al., 2020).

Capillarization is very important for the development of skin organoids. Recent studies showed that human brain organoids transplanted into the adult mouse brain developed a vasculature and incorporated into local tissues [e.g., blood–brain barrier (BBB)] (Mansour et al., 2018; Grebenyuk and Ranga, 2019). Ebner-Peking et al. reported the 3D self-assembly of adult and induced pluripotent stem cell (iPSC)-derived fibroblasts, keratinocytes, and endothelial progenitors into a novel type of floating spheroid skin organoid. Multi-cell transplant self-organization facilitates the development of skin regeneration strategies using cell suspension transplantation in combination with human platelet factors (Ebner-Peking et al., 2021). We deem that these novel platforms could be leveraged to seed skin organoids with pericytes and endothelial cells, thereby facilitating the investigation of the role of angiogenesis in skin maturation or to derive vascularized skin grafts in the future.

Single-cell RNA sequencing analysis of two strains of PSC-derived skin organoids further showed that they came from anatomically different groups of ectodermal cells. Nevertheless, no immune cells, such as Langerhans cells (LCs), were detected in these skin organoids. It is possible to improve our understanding of different cell types and states, as well as their specific pathways, by mining large datasets of single-cell gene expression profiles in this research (Lee et al., 2018; Lee et al., 2020; Lee and Koehler, 2021). A recent single-cell analysis identified new subpopulations of basal cells located at the top or bottom of the reticular dermis and reported that cell fate specification is determined by the expression of lineage-specific transcription factors (Matsumura et al., 2021). Direct reprogramming techniques can convert adult cells from one type to another. The technology of fused assembloids is ripe to build different brain regions in cerebral organoids and this scheme is expected to bring new hope for reasonable source of functional skin (Figure 1B) (Soufi et al., 2012; Iacovides et al., 2016; Qian et al., 2019).

One goal of generating skin organoids is to reconstruct fully functional skin from PSCs or ASCs *in vitro*. Research has shown that dissociated human fetal skin can reconstitute hair-bearing skin in a nude mouse model because of the specific microenvironment (Yang et al., 2014). Studies on hair follicle generation from hPSC-derived cells have relied on complex bioengineering approaches or chimeric methods using human/mouse epidermal/skin cells xenografting onto nude mice to promote skin cell development and folliculogenesis (Lee et al., 2018; Kageyama et al., 2021). The ability to induce substantial vascularization and morphological maturation in skin organoids is conducive to the maturation of skin cells to obtain an adult-like skin (Ebner-Peking et al., 2021). Skin organoids represent a platform for gaining a more in-depth understanding of skin development and formation. Nevertheless, a substantial amount of work must be undertaken to improve the skin organoid system to allow the wide-spread adoption of this technology in this field.

## The Applications of Multifunctional Skin Organoids

Currently available skin constructs are mainly skin grafts, including cultured epidermal autografts, that can be cultured from skin-derived stem cells. They can effectively support skin wound healing and are also a valuable supplement to traditional skin transplantation methods. In addition, combining biomaterials can enhance the stability and functionality of skin grafts. Nevertheless, the high manufacturing costs and lack of skin appendages (e.g., glands, hair) currently limit the therapeutic application of these products (Brockmann et al., 2018; Ebner-Peking et al., 2021). Skin organoids may provide solutions of therapeutic value and clinical applicability of skin grafts and can also be used to identify new regenerative drugs, or for gene therapy. These organoids are similar to primary skin tissues in terms of composition and structure and are easily manipulated and cryopreserved (Boonekamp et al., 2019; Diao et al., 2019). Notably, more complex biotechnologies are currently available, ranging from organoids to multi-physiological systems or organs-on-chips. Some of these models perform at least as well as animal-based models (Park et al., 2019; Horejs, 2021). The overexpression of FOXC1 is sufficient to induce the reprogramming of epidermal cells to functional sweat gland-like cells, which is beneficial for wound healing and sweat gland regeneration (Yao et al., 2019). Furthermore, recent studies have reported the use of cutaneous organ models to investigate the occurrence, development, and resistance to treatment of skin tumors, such as basal cell carcinoma, squamous cell carcinoma, melanoma, and Merkel cell carcinoma (Rebecca et al., 2020). Skin organoids could similarly be used for disease modeling applications and drug screening (Yang et al., 2020; Han et al., 2021). Alternatively, patient-sourced or gene-edited donor hiPSCs could be used to simulate inherited skin diseases. For example, genetically modified autologous epidermal cells could provide a lasting treatment for vesicular skin diseases. Several studies have shown that it is possible to correct epidermolysis bullosa, a genetic skin disorder, by CRISPR/Cas9-based targeted genome editing (Itoh et al., 2011; Hirsch et al., 2017; Jacków et al., 2019). hiPSCs can become ideal sources of cells for generating skin organoids. Thus, it is evident that being able to recapitulate skin tissues in a dish has considerably broadened the scope of skin organoid applications, including the study of human skin development, disease modeling, drug testing, investigating skin barrier biology, the development of cell/gene therapies, and toxicological assessments (Niehues et al., 2018; Lee and Koehler, 2021) (Figure 1B).

hiPSC-derived skin organoids can be instantly generated from available spheroids or assembloids (Vogt, 2021) (Figure 1B), allowing their application for high-content screening and drug testing. The push for non-animal testing as laid down in European legislation, particularly regarding European cosmetics regulation, has brought animal-free regulatory testing one step closer. Skin models, such as skin organoids containing a variety of skin structures, could deliver the accuracy required for increased applicability in predicting toxicological effects on the skin (Niehues et al., 2018; Riebeling et al., 2018). Skin organoids might provide novel options for identifying promising targets for future research on the pleiotropic effects of devastating skin injuries sustained in chemical warfare (e.g., resulting from exposure to sulfur mustard) (Rose et al., 2018). Skin organoids generated through the self-organizational abilities of stem/progenitor cells will aid in the development of new strategies targeting skin regeneration and wound healing (Lee et al., 2020).

The EU H2020 HCA Organoid project has now been launched, aiming to provide a practically useful and readily extensible initial version of the Organoid Cell Atlas within 2 years. The Organoid Cell Atlas represents a basic biology and biomedical application that will enable researchers to functionally dissect and systematically perturb human biological systems, including the skin (Bock et al., 2021). Hence, skin organoids may provide an unprecedented framework to study the molecular and cellular mechanisms of skin biology, including skin diseases, while at the same time opening numerous possibilities for identifying new patient-specific therapeutic approaches.

## Limitations

Although many challenges have been surmounted, many bottlenecks remain to be overcome, and there is still a large gap between the current state of skin organoid technology and the reality of how these organoids develop and function in the body. First, as the body’s first line of defense, the skin is equipped with an impressive array of immune cells, such as LCs. However, it is not enough to simulate the structure of skin, as excluding connective tissues, blood vessels, and immune cells does not allow to recapitulate the physiological environment of normal tissues and organs (Lee et al., 2018; Lee et al., 2020).

Secondly, potential sources of skin cells (e.g., keratinocytes) are scarce, and culturing skin cells *in vitro* is difficult with current technology (Kim et al., 2018). Cell reprogramming techniques, which can help to regenerate skin cells, are still inefficient *in vitro* (Chen et al., 2014), and the cells are prone to aging (Mahmoudi and Brunet, 2012). Thus, skin organoid culture systems need to be optimized to allow for their clinical application. Systems for the *in vitro* culture of potential cells should also continue to be optimized, while more effective reprogramming techniques, such as small molecule-induced chemical reprogramming or genetic reprogramming of genes delivered by biomaterials, should be identified. The 3D regeneration of skin organoids requires better external safety, including controllability, and internal stability, as well as the non-toxicity of the biological materials used. Additionally, whether the function of regenerated skin organoids is normal and how long they could be maintained in the body also need further investigation. Other problems that need to be overcome include the heterogeneity among cell lines and differences among batches.

## Summary and Future Perspectives

To conclude, stem and progenitor cells have great potential for future cell transplantation applications and may prove to be a sustainable alternative source of skin cells. Although the production of PSCs is time-consuming and laborious, HLA homozygous iPS cells have been proposed as an alternative way to solve this problem, in that a small number of cells can be used in large numbers of patients (Okita et al., 2011; Xu et al., 2019). In addition, 3D bioprinting technology enables a much more precise simulation of biophysical properties, including organoid size, cell number, and conformation, and modification of the latter can substantially increase starting cell numbers (Lawlor et al., 2021). Although many problems and obstacles remain, patient-derived organoids should further improve our understanding of disease and heterogeneity in patients, which may lead to personalized therapies for the treatment of a variety of diseases (Dutta et al., 2017).

The generation of skin organoids represents a new hope for skin regeneration and is expected to provide a novel scheme for the diagnosis and treatment of skin diseases. Skin organoids can be used to investigate physiological functions, such as cutaneous nerve sensation and microbiome–skin interactions, as well as for exploratory research into models of cutaneous viral–bacterial co-infections, makeup testing, and high-throughput drug screening (Figure 1B). Overall, we believe that skin organoids represent a huge breakthrough that should facilitate the advancement of both basic and clinical research.
